# The role of citric acid in denture cleansing: Effects on biofilm reduction and corrosion resistance of Co-Cr alloys

**DOI:** 10.1590/1807-3107bor-2025.vol39.070

**Published:** 2025-07-07

**Authors:** Maria Helena Rossy BORGES, Luís Fernando Bandeira MIRANDA, Samuel Santana MALHEIROS, Ayrton Geroncio SILVA, João Vicente CALAZANS, Mariana Alves dos SANTOS, Elidiane Cipriano RANGEL, Valentim Adelino Ricardo BARÃO, Bruna Egumi NAGAY

**Affiliations:** (a) Universidade Estadual de Campinas – Unicamp, Piracicaba Dental School, Department of Prosthodontics and Periodontology, Piracicaba, SP, Brazil.; (b) Universidade Estadual Paulista – Unesp, Laboratory of Technological Plasmas, Engineering College, Sorocaba, SP, Brazil.

**Keywords:** Citric Acid, Dental Alloys, Denture Cleansers, Corrosion

## Abstract

This *in vitro* study evaluated the effects of citric acid (CA) on surface properties, biofilm removal, and electrochemical performance of Co-Cr alloys compared to common denture cleansers. Co-Cr discs were divided into five groups based on the decontamination solution: NaCl 0.9% (control), Corega Tabs^®^, Periogard^®^, and 10% CA. The surface was characterized at baseline in terms of morphology, topography, and chemical and phase composition. Surface properties, including microhardness, wettability, and roughness, were assessed before and after exposure to each solution. Microbial viability, metabolic activity, and morphology of the polymicrobial biofilm were assessed after treatment to evaluate the efficacy of the decontamination solutions. Electrochemical and morphological evaluations were performed to assess the impact of each solution on the alloy’s corrosion process. No significant changes in microhardness were observed (p > 0.05). Decontamination solutions significantly increased surface hydrophilicity (p < 0.05) and roughness, though Ra values remained below the threshold for bacterial colonization. All denture cleansers significantly reduced biofilm viability compared to NaCl (p < 0.05), with no viable colonies post-treatment. The CA group showed a significant reduction in bacterial metabolic activity compared to NaCl and Periogard^®^ (p < 0.05), indicating superior biofilm disruption. Electrochemical tests demonstrated that CA maintained a stable Cr-oxide passive layer, evidenced by nobler OCP values and lower i_corr_ and corrosion rates compared to Periogard^®^ (p < 0.05). SEM images revealed pitting corrosion in all groups, except CA. These findings suggest that CA is a promising and safer alternative for denture care, offering effective antimicrobial action while preserving the electrochemical integrity of Co-Cr alloys.

## Introduction

Dental alloys play an essential role in the success of the prosthetic rehabilitation, significantly impacting patients’ oral health and overall quality of life. Cobalt-chromium (Co-Cr) alloys are among the most commonly used and versatile materials in dentistry, especially for manufacturing removable partial denture frameworks.^1^ Among their advantages, Co-Cr alloys have biocompatibility, excellent mechanical strength, and satisfactory corrosion resistance, making them safe for patients and ensuring long-term structural stability.^2^ Nevertheless, certain surface features, such as roughness and porosity, combined with patients’ oral conditions and dietary habits may facilitate biofilm accumulation.^3^ Biofilm, a structured microbial community encased in a complex extracellular matrix and often containing various Gram-positive and Gram-negative bacteria as well as fungi, can lead to several oral diseases, including prosthetic stomatitis, periodontitis and caries.^4^ Additionally, microbial biofilms on dentures can contribute to systemic infections such as endocarditis, aspiration pneumonia, and gastrointestinal infections.^5^

To prevent infection and ensure the longevity of dental prostheses, chemical cleaning is an effective strategy as it disrupts the adherence of bacterial proteins to the denture surface, thus inhibiting biofilm formation and preventing its proliferation.^6^ In addition, decontaminant solutions offer a practical cleaning option for elderly patients or those with motor or cognitive impairments.^6,7^ Currently, common denture cleansers, such as effervescent cleansing tabs and chlorhexidine-based solutions, have proven effective in reducing biofilm on denture surfaces.^7^ Corega Tabs, for example, is a widely used effervescent denture cleanser specifically designed for extra-oral use. They are commonly recommended for cleaning dental prostheses due to their proven efficacy in biofilm removal and disinfection.^8^Their antimicrobial effects are primarily attributed to the presence of active ingredients such as alkaline peroxides, which release oxygen during dissolution, disrupting bacterial and fungal biofilms by oxidative stress.^9^ Similarly, chlorhexidine, the active ingredient in Periogard, is considered the gold standard antimicrobial agent in dentistry. Its broad-spectrum efficacy against bacteria and fungi is due to its ability to disrupt microbial cell membranes, leading to leakage of intracellular components and eventual cell death.^10^ Although mouthwashes containing chlorhexidine such as Periogard are specifically formulated for intra-oral disinfection, they are also indicated to clean removable partial dentures (RPD).^11^Studies have investigated the potential effects of chlorhexidine on Co-Cr alloy in RPD, highlighting concerns about the balance between corrosion and antimicrobial activity.^11^

In light of these considerations, the ideal denture cleanser should not negatively affect the Co-Cr alloy or other denture components, such as causing surface pitting or discoloration (tarnish).^12,13^ Furthermore, the cleanser should also be easy to use, affordable, and widely accessible.^14^ Due to the limited availability and the challenges some patients face in accessing certain products, new chemotherapeutic agents that reduce biofilm formation on prostheses without compromising the mechanical integrity of dental materials are being explored.

In this context, citric acid (CA), a weak organic acid containing three carboxylic functional groups found in citrus fruits, has garnered attention as a potential alternative for denture disinfection.^15,16^ Although the precise mechanism of CA’s antimicrobial action is not fully understood, it is believed that its ability to alter pH levels allows it to freely penetrate bacterial membranes, causing structural damage to cells and, consequently, disrupting biofilm architecture.^17^ Furthermore, research has demonstrated CA’s efficacy in decreasing microbial viability while minimizing the risk of corrosion or structural degradation of metals.^18^ In addition to its antimicrobial properties, CA is biodegradable, non-toxic, and widely available, making it an eco-friendly and cost-effective option for denture care.^19^

Beyond antimicrobial effects, the impact of any decontamination agent on the electrochemical properties of the metals used in denture frameworks should be investigated.^14^ Body fluids, which contain ions and proteins, can accelerate material degradation, and the chemicals in decontamination solutions can further modify the electrochemical behavior of metal alloys, potentially compromising their long-term stability.^20^ The corrosion resistance of cobalt-based alloys primarily relies on the formation of a passive Cr_2_O_3_ oxide layer, which acts as a protective barrier.^1^ However, components such as fluoride, chlorhexidine, and alkaline peroxide, commonly found in commercial denture cleansers, can compromise the integrity of the protective oxide layer, leading to pitting corrosion—a localized form of corrosion that creates cavities in the alloy surface.^21,22^ This can result in the structural deterioration of the framework, potentially causing mechanical failure of the denture and the release of metal ions, which may have harmful biological effects.^3,20^ In this way, understanding how decontamination agents affect the corrosion resistance of Co-Cr alloys is essential for ensuring both the durability and safety of dental prostheses.

Considering the limitations of current denture cleansers, there is a clear need for alternatives that provide effective antimicrobial action without adversely affecting the corrosion resistance of Co-Cr alloys. Therefore, the aim of this study was to evaluate the efficacy of CA in removing biofilm from Co-Cr alloys and to assess its impact on the electrochemical behavior of the metal. The findings of this study will lead to a better understanding of the effects of cleansers on the Co-Cr electrochemical performance and ensure a safe indication of disinfectant agents for patients to effectively sanitize their prostheses.

## Methods

### Experimental design

**Figure 1:**
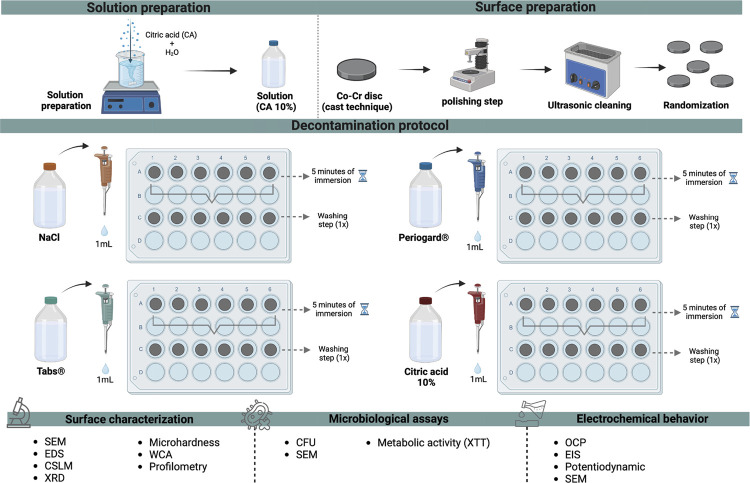
Schematic diagram of the experimental design.

This *in vitro* study was approved by the local Research and Ethics Committee (protocol number: 81499524.5.0000.5418). Informed consent was obtained from all participants, who were selected based on established criteria for good oral and general health, following an established protocol for *in vitro* biofilm formation.^23^ Co-Cr alloys discs were randomized into different decontamination solution groups (Figure 1): NaCl 0.9 % (negative control, sodium chloride solution - Sigma-Aldrich, St. Louis, MO, USA); Tabs^®^ (Corega Tabs^®^, prepared according to the manufacturer’s instructions); Periogard^®^ (0.12% chlorhexidine digluconate solution); and CA (10% citric acid solution prepared using deionized water - Dinâmica Ltd, Diadema, Brazil). For all analysis, the decontamination protocol involved immersing each sample in 1 mL of the respective solution in a 24-well plate for 5 minutes. This period was selected to maintain standardization with the immersion times of the other agents used in the study. After this period, the samples were rinsed once in distilled water.

SEM: scanning electron microscopy (n = 2/group); EDS: Energy-dispersive X-ray spectrometry (n = 2/group); CLSM: confocal laser scanning microscopy (n = 2/group); XRD: X-ray diffractometry (n = 2/group); Microhardness (n = 5/group); WCA: water contact angle (n = 5/group); Profilometry (n = 5/group); CFU: colony forming units (n = 5/group); Metabolic activity (XTT) (n = 5/group); SEM (n = 2/group); OCP: open circuit potential (n = 5/group); EIS: electrochemical impedance spectroscopy (n = 5/group); Potentiodynamic (n = 5/group); SEM (n = 2/group). Created with BioRender.com (License number: EA27ERDQQS).

### Surface preparation

The Co-Cr discs measuring 13 mm diameter x 2 mm thickness were prepared using the conventional flame cast technique and injection by centrifugation. To reduce variations such as irregular topographies that may interfere with the microbiological and electrochemical results between groups, discs were polished by standardized metallography methods (#320 and #400 SiC) (Carbimet 2, Buehler, Lake Bluff, USA) under running water using a polishing machine (Buehler, Lake Bluff, USA). After that, they were ultrasonically cleaned with deionized water (10 minutes), degreased in 70% propanol (10 minutes), and hot-air-dried.^24^

### Surface characterization

#### Structural morphology and topography

For the assessment of surface morphology, scanning electron micrographs were acquired by Scanning Electron Microscopy (SEM (JEOL JSM-6010LA) under an accelerating voltage of 15.0 kV (n = 2/group).^25^ Additionally, surface topography and three-dimensional images was assessed by 3D Confocal Laser Scanning Microscopy (CLSM) (VK-X200 series, Keyence, Osaka, Japan) at 50x magnification. Image processing was performed with the VK-Analyzer software (Keyence v3.3.0.0, Osaka, Japan) (n = 2/group).^25,26^

#### Chemical and phase composition of surface

The elemental composition of the samples was evaluated using energy-dispersive X-ray spectrometry (EDS) using (JEOL JSM-6010LA, Peabody, Massachusets, USA) (n = 2/group).^26^ Furthermore, the phase composition of the Co-Cr surface was determined by X-ray diffractometry (XRD). A diffractometer (XRD; Pana-lytical, X’Pert3 Powder, Almelo, Netherlands) was used with Cu-Ka radiation operating at 45 kV and 40 mA and a continuous speed of 0.02/s for 2 h (n = 2/group).^27^

#### Microhardness, wettability, and roughness properties

In order to understand the impact of the decontamination solutions on the mechanical and physical-chemical properties of the surface, specific analyses were performed. Microhardness can reflect alterations in the alloy’s surface integrity, which may impact its resistance to wear, deformation, and fatigue over time.^28^ To evaluate this, Vickers microhardness was measured using an indenter (Shimadzu, HMV-2 Micro Hardness Tester, Shimadzu Corporation, Kyoto, Japan) with the application of a 0.5-kgf load for 15 s.^28^ Four measurements were performed at different areas of each specimen, and the mean hardness value was calculated for each sample and group (n = 5/group). Considering that wettability is a key factor for bacterial adhesion, by evaluating wettability after immersion we can assess how the tested agents impact the material’s interaction with oral microorganisms. To this end, surface wettability was measured by water contact angle using an automated goniometer (Ram e-Hart 100e00; Ram e-Hart Instrument Co., Succasunna, USA) with deionized water via the sessile drop method (10 µL), analyzed with appropriate software (DROPimage Standard, Ram e-Hart Instrument Co.) (n = 5/group). The test was performed on dry samples immediately after the decontamination process to ensure consistent and reliable measurements.^26^ Finally, surface roughness was determined using contact profilometry (Dektak 150-d; Veeco, Plainview, USA). Parameters including Ra (arithmetic mean surface roughness), Rt (vertical distance between the highest peak and the deepest valley), Rz (height between the maximum and minimum points of the profile over the sampling length), and Rq (root mean square roughness) were recorded. Three measurements at a cut-off of 0.25 mm and speed of 0.05 mm/s (in the right, center, and left regions of the disc) were taken on each sample and then averaged (n = 5/group).^26^

## Microbiological analysis

To evaluate the antimicrobial effect of solutions, a microcosm polymicrobial biofilm model, previously established by the research group, was used to mimic the microbiome of the human oral cavity.^23^ After sterilizing the sample by ultraviolet light, the discs were coated with filtered stimulated human saliva collected from two volunteers to allow protein adsorption and promote a salivary pellicle formation simulating in vivo conditions for subsequent microbial adhesion.^29^ After 30 minutes of pellicle formation, the specimens were transferred to wells containing unfiltered human saliva adjusted to approximately 10^7^ cells/mL via spectrophotometry at 550 nm (optical density = 0.1 a.u.). Samples were incubated for 24 h (10% CO_2_ at 37◦C) to form biofilm (n = 5/group).^25^ After this period, samples were washed twice with 0.9% NaCl to remove non-adherent cells and transferred to new 24-well plates for immersion in 1 mL of the decontamination solutions for 5 minutes.^16^ After the decontamination process, discs were sonicated at 7 W for 30 s in cryogenic tubes containing 1 mL of 0.9% NaCl to detach cells from the surface.^24^ In sequence, 100 μL of the sonicated polymicrobial cell suspensions was serially diluted 6-fold in 0.9% NaCl and then plated in duplicate on Columbia Blood Agar (CBA) medium with 5% defibrinated sheep blood for counting colony-forming units (CFUs).^24^ CBA plates were incubated for 48 h (10% CO_2_ at 37◦C) and the CFUs were counted with a stereomicroscope, being the data expressed on a logarithmic scale as log_10_ CFU/mL. In addition, the XTT assay was employed to analyze the influence of decontamination protocols on remaining metabolic activity of bacterial cells. The XTT reagent (Sigma-Aldrich, St. Louis, USA) was prepared based on a previous protocol^23^ and the colorimetric changes were quantified by measuring absorbance at 492 nm using a spectrophotometer (DU 800 UV-Visible Spectrophotometer, Beckman Coulter, Inc.) (n = 5/group). Finally, for analysis of the biofilm structure and morphology after decontamination (n = 2/group), samples were fixed in glutaraldehyde, sequentially dehydrated in ethanol (30%, 50%, 70%, 90%, and 100%) and sputtered with gold for analysis by SEM (JSM5600LV, JEOL USA, Inc.) operating at 15 kV.^24^

### Electrochemical behavior

To assess the direct effects of decontamination solutions on the electrochemical behavior of the Co-Cr surface, discs were treated as described in the experimental design section and subjected to standard electrochemical tests (n = 5/group). For this, a potentiostat (Interface 1000, Gamry Instruments) connected with a three-electrode cell was used to carry out three tests: open circuit potential (OCP), electrochemical impedance spectroscopy (EIS), and potentiodynamic polarization, following our previously established protocols.^25,27^ Artificial saliva (10 mL) at 37 ± 1°C with a 6.5 pH was used as the electrolyte solution. OCP measurements were conducted for 1 hour to determine the material’s free corrosion potential. EIS was performed over a frequency range of 100 kHz to 5 mHz with an AC amplitude of ±10 mV using the OCP as the initial potential. Finally, polarization scans were conducted from -0.8 to 1.8 V at a scan rate of 2 mV/s to generate potentiodynamic polarization curves.^25^ All experimental data were processed using the Echem Analyst software (Gamry Instruments). EIS data were modeled using an equivalent circuit to determine the polarization resistance (R_p_) and capacitance (Q). The potentiodynamic polarization curves were evaluated using the Tafel extrapolation method, providing key parameters such as corrosion potential (E_corr_), corrosion current density (i_corr_), and corrosion rate.^25^ In addition, to assess potential surface morphology alterations after the corrosion process, samples were analyzed by SEM. For this, after completing the electrochemical analysis, the samples were sputtered with gold and analyzed by SEM (JSM5600LV, JEOL USA, Inc.) operating at 15 kV (n = 2/group).

## Statistical analysis

The sample size calculation was performed using G*Power software based on a significance level of 5% and a statistical power of 80%. The data were analyzed with a statistics software (SPSS v. 20.0, SPSS Inc.). The normality and homoscedasticity of all response variables were tested by the Shapiro-Wilk and Levene methods, respectively. Statistical analyses were performed using one-way ANOVA followed by Tukey’s HSD test as a post-hoc for multiple comparisons. A significant mean difference at the 0.05 level was set for all statistical tests. Final graphs were prepared using GraphPad Prism version 8.0.0 for Windows (GraphPad Software, USA).

## Results

### Initial surface properties

**Figure 2:**
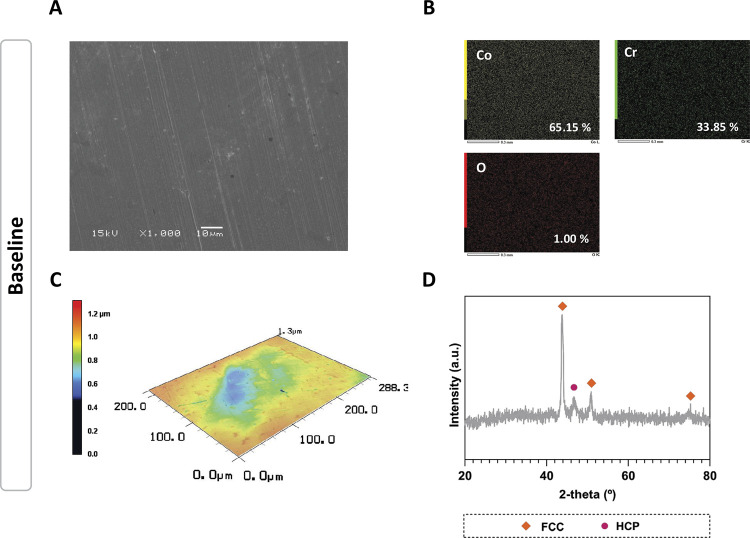
Baseline surface properties. (A) Surface morphology by scanning electron microscopy (bar = 10 μm, 1000x magnification, 15 kV). (B) EDS maps and individual elements. (C) Representative three-dimensional image by confocal laser scanning microscopy (CLSM; 150x of magnification). (D) Phase composition by X-ray diffractometry (XRD).

SEM analysis (Figure 2A) revealed that the baseline surface exhibited a smooth texture, attributed to the polishing process. Concerning the EDS maps, the chemical elements were dispersed homogeneously along the surface, with 65.15% Cobalt (Co), 33.85% chromium (Cr) and 1.00% oxygen (O) (Figure 2B). The two- and three-dimensional images obtained through CLSM corroborated the SEM findings, showing a smooth and polished surface associated with the sandpapers used during the polishing process (Figure 2C). Furthermore, the phase composition of Co-Cr surface was analyzed through X-ray diffraction. The analysis identified both the face-centered cubic (FCC) a-Co phase (ICDD card no.: 15-0806) and the hexagonal close-packed (HCP) ε-Co phase (ICDD card no.: 05-0727) (Figure 2D).^30^

### Surface characterization

**Figure 3:**
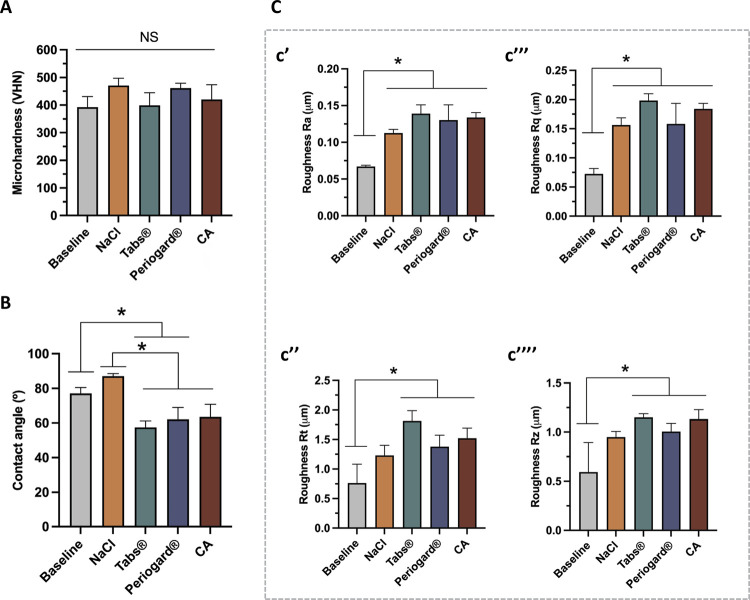
Surface properties before (baseline) and after the decontamination protocol.

To assess the effects of different decontamination solutions on Co-Cr alloys, we analyzed microhardness, surface wettability, and roughness properties. The Co-Cr surface microhardness values before and after immersion in the decontamination solutions were statistically similar (p > 0.05), suggesting that a 5 minutes immersion in 1 mL of the decontamination solutions tested did not significantly affect the microhardness of the material. Regarding the surface wettability, the water contact angle values were significantly influenced by the type of denture cleansers used. The baseline and NaCl samples exhibited water contact angles (WCA) of approximately 78 and 80 degrees, respectively. In contrast, the Tabs^®^, Periogard^®^, and CA solutions led to a significant reduction in WCA (P < .05), indicating increased surface hydrophilicity. Notably, although the CA solution also reduced WCA, this effect was less pronounced than the reductions observed with Tabs^®^ and Periogard^®^ when compared to baseline measurements. Similarly, the denture cleansers increased the surface roughness parameters (Figure 3C), showing significant differences in Ra, Rq, Rt, and Rz values (Figure 3c’, c’’, c’’’, c’’’’) across the groups compared to the baseline samples (p < .05). This suggests that the different cleansing treatments altered the surface texture of the materials to different extents.

(A) Surface microhardness. (B) Wettability as indicated by water contact angle. (C) Surface roughness, including (c’) average surface roughness (Ra), (c’’) maximum height of the profile (Rt), (c’’’) root mean square roughness (Rq), (c’’’’) average maximum height of the profile (Rz).

*p < 0.05, by one-way ANOVA and Tukey’s HSD test. The error bars indicate standard deviations. NS, not significant.

### Microbiological analyses

**Figure 4:**
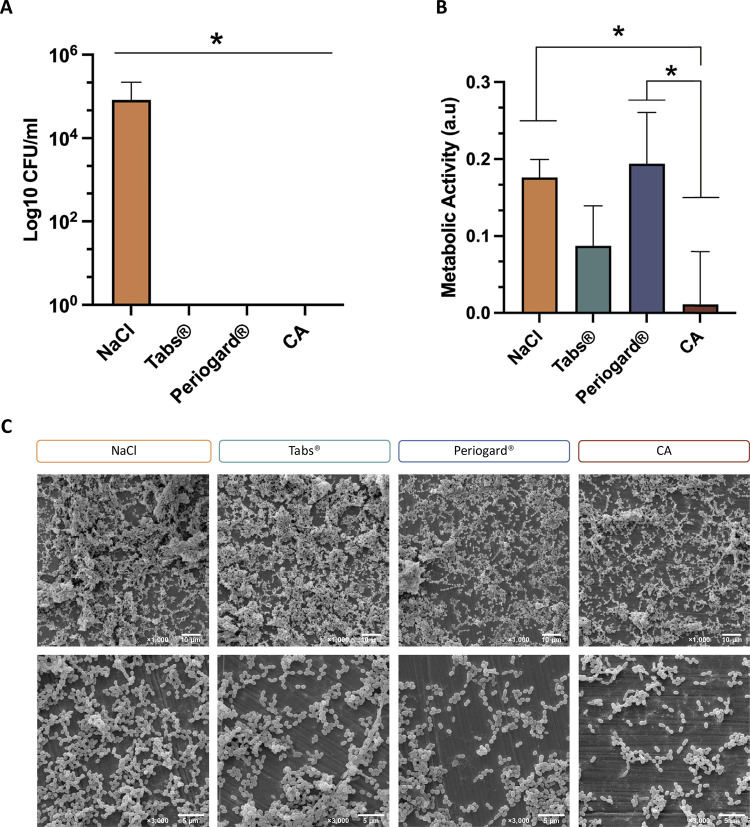
Microbiological assays and biofilm characteristics after decontamination protocols. (A) Polymicrobial biofilm counts (CFU/mL), (B) metabolic activity measured by XTT assay, and (C) biofilm morphology observed through SEM imaging (1000x and 3000x magnification).

Microbiological analysis revealed that all decontamination solutions, except for the control NaCl, provided a significant microbicidal effect against the polymicrobial biofilms (Figure 4A) (p < .05). In addition, the XTT assay (Figure 4B) showed that the CA solution significantly reduced biofilm metabolic activity compared to NaCl and Periogard^®^ (p < 0.05). SEM imaging of the biofilm architecture showed a complex extracellular matrix, consisting of bacteria with different shapes, including cocci and bacilli. While all solutions significantly reduced microbial viability when compared to NaCl, their effectiveness in removing non-viable biofilm from Co-Cr surface was less pronounced. However, the SEM images showed that the CA group had a subtle reduction in biofilm coverage compared to the other treatments, corroborating the findings from the metabolic activity analysis.

*p < 0.05, by one-way ANOVA and Tukey’s HSD test. The error bars indicate standard deviations.

### Electrochemical behavior and morphological features

**Figure 5:**
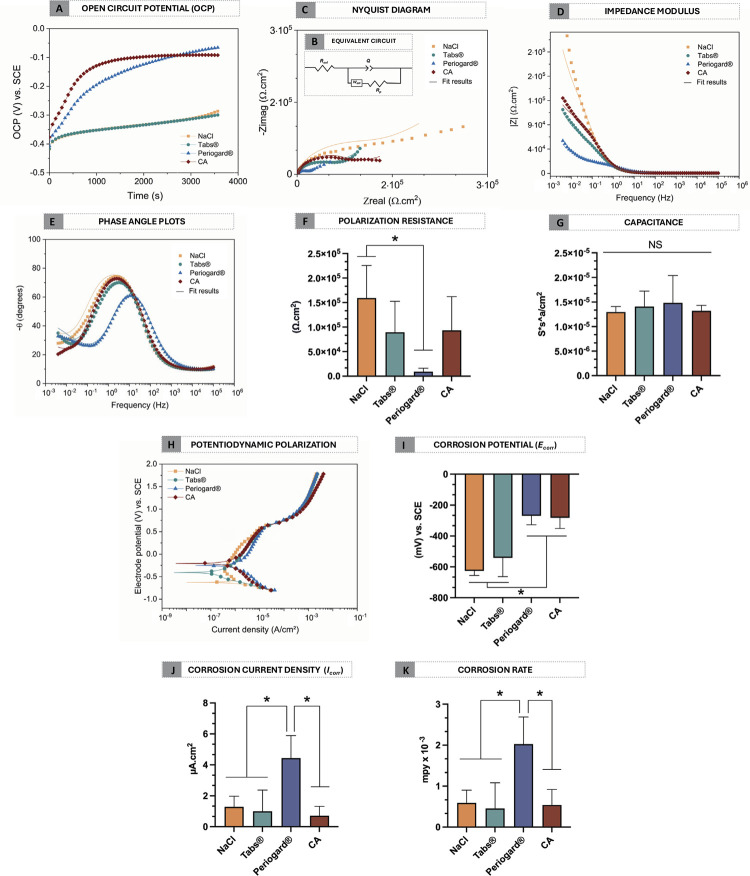
Electrochemical performance of Co-Cr alloys in artificial saliva as a function of different decontamination solutions. (A) Representative curve of open circuit potential (OCP) evolution (in V vs. SCE – saturated calomel electrode). (B) Equivalent electric circuit applied for electrochemical impedance spectroscopy (EIS) data, consisting of Rsol (resistance of the solution), Rp (polarization resistance), Q (constant phase element, CPE), and Wdff (Warburg element), (C) representative Nyquist diagrams, (D) impedance modulus, and (E) phase angles of EIS assay. Electrical parameter values such as (F) polarization resistance and (G) capacitance are collected from EIS (goodness of fit on the order of 10−3). (H) Potentiodynamic polarization curves (in V vs. SCE). (I) Corrosion potential (Ecorr), (J) Corrosion current density (icorr) and (H) corrosion rate values were obtained.

Regarding electrochemical outcomes, Figure 5A illustrates the evolution of OCP for all groups in artificial saliva at 37°C over time. The CA group showed higher and stable OCP values (-752.30 ± -182.60 mV) compared to the NaCl (-835.30 ± -569.20 mV) and Tabs^®^ (-833.00 ± -597.60 mV) groups.

*p < 0.05, by one-way ANOVA and Tukey’s HSD test. The error bars indicate standard deviations.

In addition, EIS was carried out to assess the corrosion kinetics of both control and experimental groups. The equivalent circuit employed for simulating the Co-Cr surface’s electrical parameters (Figure 5B) consisted of R_sol_ (resistance of the solution), R_p_ (polarization resistance), Q (constant phase element, CPE), and W_dff_ (Warburg diffusion element). The obtained chi-square (χ^2^) values were of the order of 10^-3^, which indicates great agreement between the experimental and simulated EIS data. The Nyquist diagram (Figure 3C) displayed the relationship between real (Z_real_) and imaginary (Z_imag_) impedance components, where the NaCl and CA groups had the largest Nyquist arcs, confirming improved protective behavior of the Co-Cr alloy. Furthermore, the variation in impedance as a function of frequency was showed by impedance modulus diagram (Figure 5D). The NaCl and CA groups showed higher impedance values at low frequencies. In contrast, the Periogard^®^ group exhibited the lowest impedance values, suggesting poorer corrosion resistance. The phase angle plot (Figure 5E) further corroborated these findings, showing similar values across the groups, with the Periogard^®^ group exhibiting lower phase angle values, indicating reduced electrochemical stability. Additionally, after exposure to the Periogard^®^ solution, the R_p_ of the Co-Cr alloy decreased (p < 0.05) (Figure 5F), suggesting a reduction in the protective barrier. Concerning capacitance values, no statistically significant difference was identified between the groups (Figure 5G).

Finally, potentiodynamic polarization curves (Figure 5H) revealed that the CA group shifted toward more electropositive potentials compared to the Tabs^®^ group and exhibited lower corrosion potential than the Tabs^®^ group (p < 0.05) (Figure 5I). Electrochemical parameters also showed that the CA group had significantly lower corrosion current density (i_corr_) (Figure 5J) and corrosion rates (Figure 5K) compared to the Periogard^®^ group (p < 0.05), indicating a slight improvement in electrochemical performance.

**Figure 6:**
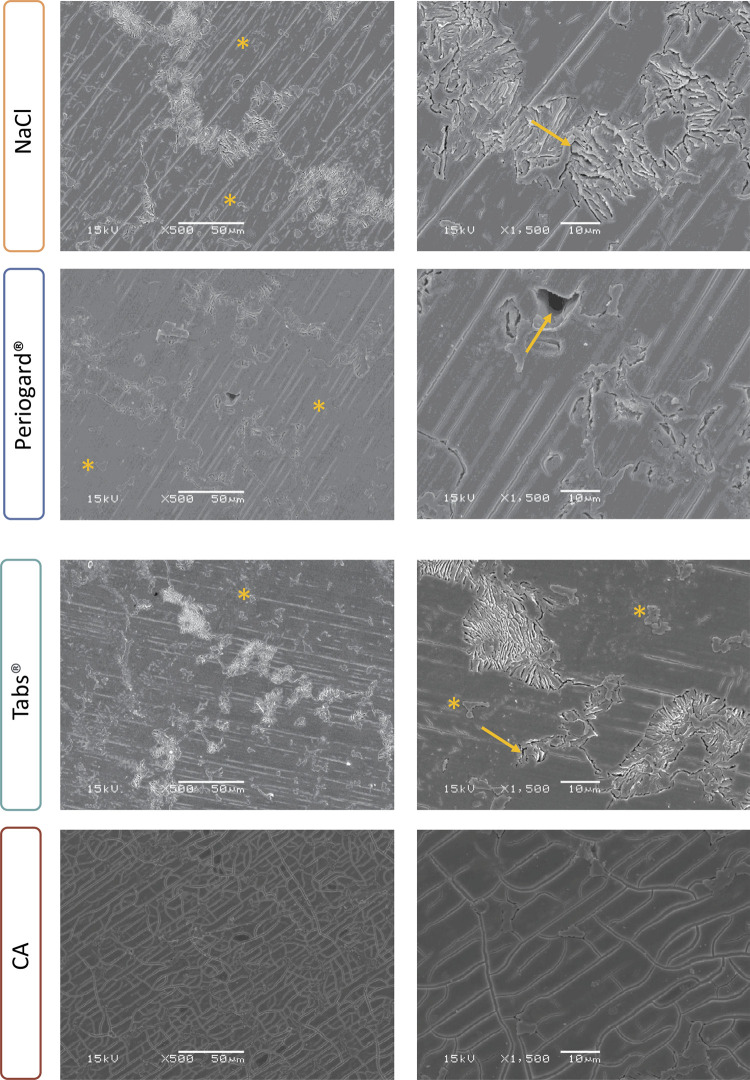
SEM micrographs (magnification of 500x and 1500x) of Co-Cr alloy after corrosion analysis and immersion in NaCl, Tabs®, Periogard®, and CA.

SEM analysis of the Co-Cr surfaces revealed clear signs of corrosion attack (Figure 6). The NaCl, Periogard^®^, and Tabs^®^ groups showed significant microstructural changes, including corrosion products (marked with asterisks) and pitting corrosion (cavity formation, marked with arrows). In contrast, the CA group exhibited interdendritic or intergranular corrosion, indicating a process that progresses preferentially along grain boundaries.

Asterisks indicate chemical deposits and arrows indicate pitting corrosion areas.

## Discussion

The decontamination solutions evaluated in this study demonstrated significant effects on the variables analyzed, including surface properties, antimicrobial activity, and electrochemical behavior, highlighting their diverse impact on the structural and functional integrity of the Co-Cr alloy surfaces.

Previous studies have demonstrated that immersing dentures in cleansing agents can increase the surface roughness of both the denture acrylic and metal framework.^13,31^ For instance, studies comparing the retention of Candida albicans on smooth and rough acrylic resins found a higher number of cells adhered to roughened surfaces.^32^ However, other research has reported minimal effects of decontamination agents on the morphological features of denture components, suggesting that surface alterations may vary depending on the type of agent, the exposure duration, and the material properties of the prosthesis.^13^ Overall, while all decontamination solutions tested showed a slight increase in roughness after immersion, it is important to note that Ra values remained below 0.2 µm, a threshold considered low enough to minimize the risk of bacterial adhesion.^33^

Surface roughness also plays a significant role in the wettability of materials. It is important to highlight that, although the cleaning solution might not directly etch the surface, its interaction with the passive oxide layer of cobalt-chromium alloys could alter the layer’s stability or uniformity, potentially resulting in microscale pitting.^34^ Such subtle changes may contribute to an increase in surface roughness, even in the absence of significant chemical reactivity.^34^ Furthermore, as noted by Uhorchuk et al.,^35^ surface roughness affects the wetting characteristics, as evidenced by changes in the static contact angle. This observation is consistent with Cassie-Wenzel theory and Nishioka, which states that an increase in surface roughness leads to an increase in surface area, thereby enhancing the solid’s affinity for liquids.^31,36^ In our study, we observed that as the surface roughness of the Co-Cr alloy increased, the contact angle decreased, indicating increased surface hydrophilicity. In addition to this mechanism, we believe that chemical interactions between the cleanser solutions and the metallic surface can further influence this behavior.^37,38^ For instance, CA can promote the removal of surface contaminants by carboxylation and the hydrolysis of ester bonds, which introduce polar groups and consequently increase surface hydrophilicity.^37^ Similarly, Tabs^®^, which contains alkaline peroxide, may promote surface cleaning by facilitating the breakdown of organic matter and enhancing wettability through oxidative processes.^38^ However, it is also possible that residual agents from the decontamination solutions, even after washing, contributed to the observed changes in wettability.^39^ These residues may form a thin film or chemically alter the surface, potentially affecting the contact angle.^38^ For example, polar functional groups left behind from CA or oxidation products from Tabs^®^ could further enhance hydrophilicity.^39^ This highlights the importance of thoroughly rinsing prostheses after decontamination to minimize the effects of residues.^37,38^ In a clinical context, the improved hydrophilicity of the Co-Cr alloy following decontamination could positively impact the interaction of the metallic framework with the oral cavity. Given the increased hydrophilicity, residual food particles, plaque, and stains could be more easily removed with gentle water pressure, enhancing oral hygiene.^40^

Concerning antimicrobial efficacy, the CA solution demonstrated a considerable microbicidal effect, as evidenced by the absence of viable colonies, noticeable reduction in biofilm coverage, and a significant reduction in bacterial metabolic activity. This indicated that CA achieves microbial killing comparable to common denture cleansers, further reinforcing its potential as an effective alternative for clinical use.^16^ However, while the CFU results provide a quantitative and reliable measure of microbial viability, showing equivalent reductions across all tested agents, CA exhibited a distinct advantage in the metabolic analysis, with the lowest metabolic activity compared to NaCl and Periogard. This discrepancy between CFU and metabolic assay results may be attributed to several factors. One explanation lies in the presence of sub-lethally damaged cells or those in a viable but non-culturable (VBNC) state. In this state, bacterial cells maintain metabolic activity but are unable to replicate.^41^ The VBNC state is a bacterial survival strategy under stress conditions, allowing cells to persist despite antimicrobial treatments. This phenomenon can explain the residual metabolic activity detected in XTT assays, particularly in groups treated with NaCl and Periogard, even as CFU results indicate significant bacterial death.^42^ Additionally, the protective role of the biofilm matrix further complicates this relationship. The biofilm matrix acts as a barrier, shielding bacterial cells from complete exposure to antimicrobial agents. This barrier may delay the penetration of decontaminants, allowing some bacterial cells to retain metabolic activity immediately after treatment, as detected in the XTT assay.^43^ However, over time, as reflected in the CFU analysis, antimicrobial agents like CA may successfully penetrate the matrix, causing greater bacterial degradation and inhibiting replication. These findings suggest that CA not only causes microbial killing comparable to commonly used denture cleansers but also exhibits distinct advantages in reducing metabolic activity. Such results underscore CA’s potential benefits in clinical settings where rapid and effective disinfection is critical. This is particularly relevant in the case of removable partial dentures, where the metal framework is in close contact with teeth. The metal structure can serve as a reservoir for microorganisms, which can detach from biofilms and spread to other areas of the oral cavity.^44^ This increases the risk of secondary infections, such as caries and periodontitis, which could compromise both the remaining teeth and the overall success of the prosthetic rehabilitation. In general, the antimicrobial action of CA is primarily attributed to its ability to significantly lower the pH of extracellular sites.^17,18^ This acidification disrupts the hydrogen ion gradient across bacterial membranes, affecting both membrane permeability and metabolic processes.^17^ Moreover, CA’s broad-spectrum activity also extends to fungi, as evidenced by its effectiveness against Candida albicans, a significant opportunistic pathogen associated with prosthetic stomatitis.^45^

Regarding the impact of the solutions on electrochemical behavior, the corrosion of Co-Cr alloys not only impair physicochemical properties but also negatively affects biocompatibility.^46^ Furthermore, the surface microstructure and mechanical resistance are closely tied to an alloy’s electrochemical performance.^20^ To ensure clinically relevant conditions, artificial saliva was used as the electrolytic solution in this study.^16^ After decontamination, the prostheses are inserted into the oral cavity and come into contact with saliva. Performing electrochemical analyses after the extraoral cleaning step replicates this real-life sequence, allowing for an accurate evaluation of Co-Cr alloy corrosion behavior under conditions that closely mimic clinical use.

In the present study, the OCP, EIS, and potentiodynamic tests revealed key insights into the corrosion behavior of Co-Cr alloys under different decontamination solutions. A particularly notable finding was the stable electrochemical behavior observed in the CA group, characterized by nobler potential values and a near-constant potential over time. This stability is closely linked to the proper formation of a stable Cr-oxide passive layer and can be attributed to the minimal volumetric difference between chromium and its oxide, which enhances the mechanical stability of the surface.^1^ This mechanical integrity of the passive film prevents the formation of cracks and fractures, thereby promoting the sustained passivity and long-term corrosion resistance of Co-Cr alloys.^47^

Concerning EIS analysis, higher values of impedance at low frequencies were observed for the NaCl, Tabs^®^, and CA groups, suggesting a suitable electrochemical behavior and the formation of a stable oxide film on the metallic surface.^25^In general, the CA group exhibited a behavior comparable to that of the control group (NaCl), indicating that CA did not negatively affect the corrosion resistance of the Co-Cr alloy. This highlights the harmless effect of CA on the electrochemical stability of the material, as the passive Cr oxide layer remained effective in maintaining its protective properties.^20,22^

Potentiodynamic measurements were performed in order to verify the local corrosion, which is related to the breakdown of the passive layer. The Tabs^®^ group exhibited lower negative E_corr_ values, indicating lower corrosion resistance compared to the CA and Periogard^®^ groups.^25,27^ This outcome may be attributed to the chemical composition of the effervescent tablets and the oxygen bubbles generated during dissolution. When the sodium perborate in the tablets comes into contact with water, an alkaline peroxide solution is produced, releasing oxygen ions.^20,38^ These ions penetrate the surface, leading to a breakdown of the protective barrier and negatively impacting corrosion potential.^38^ Notably, Periogard^®^ generated the highest values of i_corr_ and corrosion rate among groups, suggesting the higher degree of passive film dissolution. This mechanism is likely driven by chloride ions (Cl-) in Periogard^®^, which can be easily adsorbed onto the passive film, leading to its breakdown.^21^ The adsorption of Cl^-^ ions may accelerate oxidative stress, ion penetration, and pitting corrosion. Additionally, the Cl^-^ ions displace oxygen, forming soluble metal-anion complexes, which further reduce the stability of the passive film and increase the corrosion rate.^21^

In this context, the balance between microbial inhibition and the risk of pitting corrosion is critical and influenced by the chemical composition of the cleanser solutions and their ability to form complexes with metal ions. Notably, SEM images after exposure to NaCl, Periogard^®^, and Tabs^®^ solutions revealed significant microstructural changes, including alloy dissolution, the formation of pits, and corrosion products, indicating a high level of corrosion.^48^ These pits act as stress concentrators that penetrate deep into the metal, ultimately weakening its structural integrity and potentially leading to mechanical failure, particularly in high-stress environments such as dental prostheses.^48^ Moreover, given that the corrosion rate analysis simulates the equivalent of one year of metal exposure, this form of corrosion can lead to the release and dissolution of cobalt from the alloy surface, which may compromise the biocompatibility of the material and induce hypersensitivity or allergy.^49^ Additionally, the deterioration leaves behind chromium oxide (Cr^3^), a key component crucial for maintaining the alloy’s corrosion resistance.^20,21^ Conversely, the “interdendritic” or “intergranular corrosion” observed in CA groups occurs along the grain boundaries or dendritic structures of the metal. This could be attributed to a more suitable concentration of CA and the presence of citrate ions, which might form more stable and protective complexes with the metal surface.^50^ In light of these findings, along with the accompanying electrochemical data, the CA solution appears to be a less aggressive alternative that effectively preserves the integrity of the Co-Cr alloy’s surface layer without jeopardizing its antimicrobial efficacy.

Therefore, the influence of decontamination solutions on surface characteristics, antimicrobial performance, and corrosion resistance of Co-Cr alloy is clinically significant, as it directly affects the alloy’s performance in the oral cavity, including its durability and overall effectiveness in rehabilitation treatments.^22^ In this sense, CA appears as an excellent option as a decontamination agent for denture care, offering protection against degradation of the Co-Cr alloy. Nevertheless, our ﬁndings should be carefully interpreted since long-term exposure was not evaluated to confirm CA safety and superiority. A limitation of this study is that the physical and chemical properties of the substrates were evaluated after the exposure to the decontamination solutions without prior biofilm formation. While this approach focuses on the solutions’ direct effects, it does not account for the combined effects of biofilm and decontamination, which should be addressed in future studies. Furthermore, the association of CA with mechanical cleaning is particularly relevant, as it could enhance biofilm removal while causing surface wear. This wear may influence the electrochemical and structural properties of the alloys over time, highlighting the need for further investigation in future studies to fully understand these effects.

## Conclusion

Based on the findings of this study, the use of CA as a cleaning agent for dental prosthesis represents a promising balance between effective microbial control and the preservation of the Co-Cr alloy’s structural integrity. CA positively alters the characteristics of the Co-Cr surface, increasing its hydrophilicity without negatively impacting its microhardness, roughness and electrochemical behavior. Given the critical importance of preserving both surface properties and corrosion resistance of the denture framework, CA proves to be a highly effective decontaminant for denture care, successfully reducing bacterial viability without compromising the alloy’s electrochemical performance.

## Data Availability

The contents underlying the research text are contained in the manuscript.
